# Frequency and Hearing Loss Phenotypes of *OPA1* Variants in a Cohort of 18,475 Patients with Hearing Impairment

**DOI:** 10.3390/genes17030341

**Published:** 2026-03-19

**Authors:** Masayuki Kawakita, Hideaki Moteki, Shin-ya Nishio, Yumiko Kobayashi, Mika Adachi, Takayuki Okano, Hiroshi Yamazaki, Jun Nakayama, Shinya Ohira, Takashi Ishino, Yutaka Takumi, Shin-ichi Usami

**Affiliations:** 1 Department of Otorhinolaryngology-Head and Neck Surgery, Shinshu University School of Medicine, Matsumoto 390-8621, Japan; rmchampionmkred@shinshu-u.ac.jp (M.K.); takumi@shinshu-u.ac.jp (Y.T.); 2 Department of Hearing Implant Sciences, Shinshu University School of Medicine, Matsumoto 390-8621, Japan; moteki@shinshu-u.ac.jp (H.M.); nishio@shinshu-u.ac.jp (S.-y.N.); 3 Department of Otolaryngology, Aizawa Hospital, Matsumoto 390-8510, Japan; 4 Department of Clinical Genetics, Iwate Medical University, Morioka 020-8505, Japan; ymkobaya@iwate-med.ac.jp; 5 Department of Otolaryngology-Head and Neck Surgery, Tohoku University Hospital, Sendai 980-8574, Japan; mika.adachi.a5@tohoku.ac.jp; 6 Department of Otolaryngology-Head and Neck Surgery, Fujita Health University, Toyoake 470-1192, Japan; takayuki.okano@fujita-hu.ac.jp; 7 Department of Otolaryngology-Head and Neck Surgery, Graduate School of Medicine, Kyoto University, Kyoto 606-8507, Japan; h_yamazaki@ent.kuhp.kyoto-u.ac.jp; 8 Department of Otorhinolaryngology-Head and Neck Surgery, Shiga University of Medical Science, Otsu 520-2192, Japan; jfukui@belle.shiga-med.ac.jp; 9 Department of Otorhinolaryngology, Shonan Kamakura General Hospital, Kamakura 247-8533, Japan; shinya.oohira@med.toho-u.ac.jp; 10 Department of Otorhinolaryngology, Head and Neck Surgery, Graduate School of Biomedical and Health Sciences, Hiroshima University, Hiroshima 734-8553, Japan; tishino@hiroshima-u.ac.jp

**Keywords:** *OPA1*, syndromic hearing loss, dominant optic atrophy, auditory neuropathy spectrum disorder, cochlear implant

## Abstract

Background/Objectives: The *OPA1* gene encodes a dynamin-related GTPase essential for mitochondrial fusion. Variants in *OPA1* are a major cause of autosomal dominant optic atrophy (DOA). A subset of DOA patients exhibits hearing loss, often manifesting as auditory neuropathy spectrum disorder (ANSD). In this study, we aimed to describe the frequency of *OPA1*-related hearing loss in a large cohort of patients with hearing loss and to explore the genotype–phenotype correlations and appropriate interventions. Methods: A total of 18,475 Japanese patients with hearing loss were recruited. Targeted massively parallel sequencing of 158 deafness-related genes was performed, and individuals with *OPA1* variants were identified. Clinical data, including age of onset, audiological findings, and systemic features, were retrospectively reviewed. Results: Ten individuals from eight independent families carrying *OPA1* variants were identified. Three variants were classified as pathogenic or likely pathogenic, while five were variants of uncertain significance. Hearing loss was typically post-lingual in onset and progressive, with predominantly mild-to-moderate severity. Missense variants tended to be associated with DOA-plus phenotypes and ANSD. Five patients obtained only limited benefit from hearing aids, whereas one patient who received a cochlear implant achieved good speech perception. Conclusions: *OPA1* is a rare causative gene for hearing loss and is frequently associated with the ANSD phenotype. Affected individuals exhibited phenotypic heterogeneity, which may reflect incomplete penetrance or the influence of mitochondrial DNA-related factors.

## 1. Introduction

Congenital sensorineural hearing loss (HL) is one of the most common sensory disorders, occurring in one in 700–1000 newborns, with about 60% of cases due to genetic causes [1]. A recent epidemiological survey in Japan involving 153,913 newborns revealed that 1.62 per 1000 newborns had HL [2]. Among the genetic causes of HL, ~80% are autosomal recessive (AR), and ~15% are autosomal dominant (AD). In addition, X-linked or mitochondrial inheritance is rare [3]. Interestingly, AR variants generally cause congenital, non-progressive HL, whereas AD variants typically lead to post-lingual onset and progressive HL [1].

The *OPA1* gene, located on chromosome 3q28-29, encodes a dynamin-related mitochondrial inner membrane GTPase that plays an essential role in mitochondrial fusion, cristae integrity, and oxidative phosphorylation [4]. *OPA1* has been identified as the causative gene for autosomal dominant optic atrophy (DOA), accounting for approximately 90% of DOA cases [5]. DOA is characterized by a slowly progressive bilateral visual loss occurring in childhood [6]. Beyond isolated optic atrophy (OA), increasing evidence indicates that pathogenic variants in *OPA1* also contribute to HL. *OPA1*-related HL has been increasingly recognized as a component of DOA-plus syndromes, which encompass OA, sensorineural HL, myopathy, peripheral neuropathy, and other systemic manifestations [7,8,9]. Given the high metabolic demands of cochlear hair cells and spiral ganglion neurons, the mitochondrial dysfunction, oxidative stress, and impaired bioenergetics caused by *OPA1* variants contribute to auditory dysfunction [10]. *OPA1* variants also lead to an auditory neuropathy spectrum disorder (ANSD) phenotype characterized by abnormal auditory brainstem responses (ABR) and auditory steady-state responses (ASSR) with preserved distortion product otoacoustic emissions (DPOAEs) [11,12]. This pattern reflects preserved outer hair cell function with impaired auditory nerve conduction, suggesting a postsynaptic auditory deficit [13]. ANSD refers to a range of hearing impairments characterized by poor speech perception despite relatively preserved pure-tone thresholds [14]. Most previous reports have focused on the clinical phenotypes of OA, and the detailed characteristics of HL, such as HL type, severity, hearing deterioration rate, and outcomes of hearing aids (HAs) and cochlear implantation (CI), remain unclear.

In this study, we aimed to describe (1) the frequency, (2) the auditory phenotypes including ANSD, (3) the genotype–phenotype correlations, and (4) the interventions for HL associated with *OPA1* variants in a large patient cohort.

## 2. Materials and Methods

### 2.1. Subjects

This study was conducted as a part of a large cohort study involving 18,475 samples from 130 collaborating institutions, obtained through social health insurance-based genetic testing for the diagnosis of HL patients. Thus, our cohort was mainly composed of affected individuals. The earlier phase results of this project for 10,047 HL patients analyzed using a 63-gene panel have been reported previously [15]. Since that report, the study has continued to expand in both the number of tested individuals and the number of target genes. In the present study, we analyzed 18,475 individuals with an updated 158-gene panel that includes the *OPA1* gene to clarify the detailed characteristics of *OPA1*-related HL. Targeted resequencing analysis was performed using the Ion AmpliSeq platform (ThermoFisher Scientific, Waltham, MA, USA). For this study, we selected the patients carrying *OPA1* candidate variants from this cohort. A retrospective chart review was conducted to obtain the clinical information of *OPA1*-related HL patients. Written informed consent was obtained from all 18,475 participants and from the next of kin, caretaker, or guardian when the participants were minors or children. The study protocol was approved by the Shinshu University Ethics Committee as well as the ethics committees of each participating institution.

### 2.2. Variant Analysis

Sequencing libraries were prepared using an Ion AmpliSeq Custom Panel (ThermoFisher Scientific, Waltham) for 158 causative genes associated with non-syndromic or syndromic HL with the Ion AmpliSeq Library Kit 2.0 (ThermoFisher Scientific), according to the manufacturer’s protocol [16]. After the amplicon libraries were prepared, sequencing was performed using the Ion S5 plus system with the Ion 540 Kit-Chef and Ion 540 Chip Kit (ThermoFisher Scientific), according to the manufacturer’s procedure. The Torrent Mapping Alignment Program ver. 5.16. was used to align the sequence data against the human genome sequence (build GRCh37/hg19). Following sequence mapping, the DNA variants were identified using the Torrent Variant Caller plug-in program ver. 5.16. After variant detection, their effects were assessed using the ANNOVAR software ver. 2020-06-08 [17]. The detected variants were filtered based on the following criteria: (1) protein-affecting variants (including the missense, nonsense, insertion/deletion, and splicing variants) and (2) a minor allele frequency of less than 1% in several control databases (including the 1000 Genome Database [18], The Genome Aggregation Database ver 4.1 [19], the human genetic variation database (dataset for 1208 Japanese exome variants) [20], the 59,940 Japanese genome variation database (ToMMo 60KJPN) [21], and the 333 in-house Japanese controls with normal hearing). Filtering was performed with the original database software described in our previous paper [22]. Sanger sequencing was used to validate the identified candidate variants and to conduct family segregation analysis where applicable. We also performed copy number analysis using the read depth data for all 158 genes obtained from the NGS analysis, according to the methods described in our previous paper [23]. For missense variants, functional in silico prediction software, including Sorting Intolerant From Tolerant (SIFT), Polymorphism Phenotyping v2 Human Variation (PP2 HVAR), MutationTaster, Combined Annotation Dependent Depletion (CADD), and Rare Exome Variant Ensemble Learner (REVEL) were used in the ANNOVAR software. For splice-site variants, SpliceAI ver 1.3 and dbscSNV (ADA) in the ANNOVAR software were employed to predict the potential impact on splicing. The pathogenicity of the identified variants was evaluated according to the American College of Medical Genetics (ACMG) criteria [24], with expert specification from the ClinGen HL Clinical Domain Working Group [25]. Candidate variants were selected among the identified variants based on the following criteria: (1) previously reported pathogenic or likely pathogenic variants with no conflicting evidence; (2) novel variants predicted to be pathogenic or likely pathogenic; and (3) variants of uncertain significance (VUS) that remained as the sole candidate after filtering, with no other candidate variants observed in the other 157 genes and without any conflicting evidence. In brief, we evaluated the remaining candidate variants after the filtering procedure. When no alternative genetic diagnosis that provided a better explanation than the *OPA1* variant was identified, we employed the VUS as the candidate variant. For example, candidate variants were excluded when (1) only a heterozygous variant was detected in genes associated with autosomal recessive inheritance or (2) the variant had “benign” supporting evidence (e.g., the *in silico* prediction score supported a “benign” effect onto protein function or the minor allele frequency in the control database was over 0.0002 in autosomal dominant inheritance genes).

### 2.3. Clinical Evaluations

We retrospectively collected detailed clinical data including (1) the presence of OA, age at evaluation, age at onset of HL, and self-reported progressive worsening of hearing; (2) pure-tone audiometry; (3) DPOAEs; (4) ABR or ASSR; and (5) use of hearing devices and outcomes from medical charts. HL severity was evaluated based on pure-tone audiometry. Audiometric thresholds at four frequencies (0.5, 1, 2, and 4 kHz) were averaged to calculate the pure-tone average (PTA). PTA was categorized as mild (>25 dB and ≤40 dB HL), moderate (>40 dB and ≤70 dB HL), severe (>70 dB and ≤90 dB HL), or profound (>90 dB HL). The audiometric configurations were classified as flat, low-frequency HL, mid-frequency HL, and high-frequency HL as reported previously [26]. ANSD was defined as abnormal or absent ABR and/or severe-to-profound HL in ASSR, with preserved DPOAE responses in this study.

To assess the degree of hearing impairment, the better-ear PTA was employed. To assess the progression of HL, we analyzed the relationship between age and better-ear PTA across all available audiometric data points. A simple linear regression model was fitted with better-ear PTA as the dependent variable and age as the independent variable, using ordinary least squares. The regression equation, slope, intercept, and coefficient of determination (R^2^) were extracted from the fitted model, and a 90% confidence interval for the regression line was estimated. Visualization was performed using Python (version 3.10) with the matplotlib and statsmodels packages and included scatter plotting, the fitted regression line, and confidence interval shading.

The outcomes of interventions (HA or CI) were evaluated by chart review. Speech perception in the CI recipients was evaluated using the iPad-based Japanese speech perception test (iCI-2004) [27].

## 3. Results

### 3.1. Detected Variants

We identified eight *OPA1* variants, including one pathogenic variant, two likely pathogenic variants, and five variants of uncertain significance (VUS) (Table 1). Four of the eight variants were novel, comprising one missense variant and three splice-site variants. The minor allele frequencies of all variants were below 0.0001% in the aforementioned databases. Three splice-site variants were predicted to result in a deterioration of splicing in both SpliceAI and dbscSNV software. According to ACMG guidelines, all novel variants were classified as VUS. Segregation analysis was available only for selected families, as DNA samples were not available from all relatives. In addition, we could not perform RNA-Seq or RT-PCR analysis for splice site variants because this study was conducted retrospectively.

### 3.2. Clinical Features of Patients with OPA1 Variants

**Figure 1:**
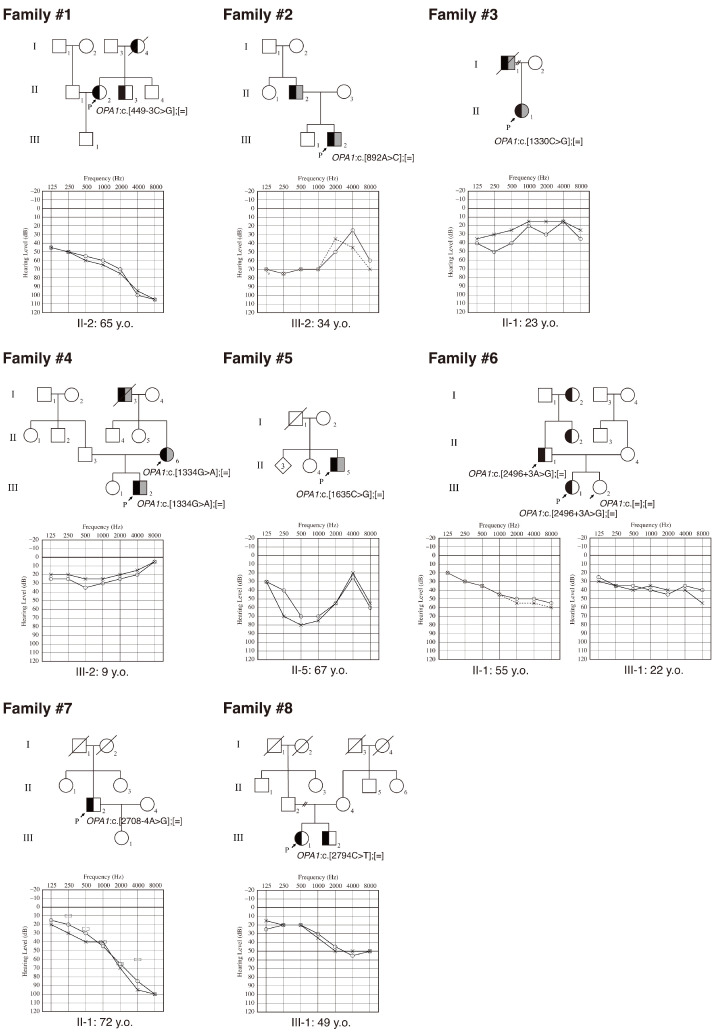
Pedigrees and audiograms of the eight families carrying *OPA1* variants. In the pedigrees, the left half of each symbol shaded in black indicates hearing loss, and the right half shaded in gray indicates optic atrophy. Arrows with “P” indicate the proband. Genetic findings of tested individuals are shown in the pedigree. Age at the time of genetic and audiometric testing is provided beneath each audiogram.

We identified ten affected individuals from eight independent families, each carrying a distinct *OPA1* variant, including four previously reported variants (Figure 1, Table 2) [28,29,30,31]. Their clinical characteristics are summarized in Table 2. Four individuals carrying missense variants, JHLB-2582, JHLB-7354, the mother of JHLB-7354, and JHLB-11374, presented with OA. The age at onset of HL ranged from 6 to 60 years, and four individuals, including JHLB-1912, the father of JHLB-4064, JHLB-16179, and JHLB-16789, first noticed HL in adulthood. Six of the eight patients demonstrated progressive HL, and all exhibited bilateral HL. The audiometric configurations consisted of a flat pattern in two, a sloping high-frequency HL pattern in four, a low-frequency HL pattern in two, and a mid-frequency HL pattern in one. Most patients had mild-to-moderate HL: two had mild HL, four had moderate HL, one had severe HL, and two had normal better-hearing ear PTA.

**Figure 2:**
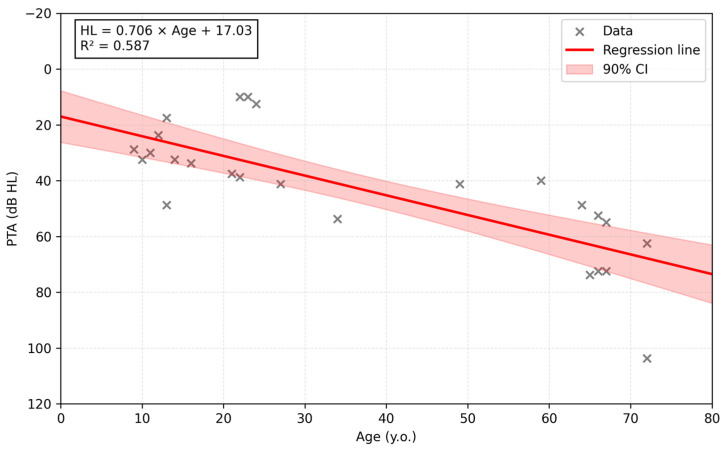
Age-related progression of better-ear hearing levels in individuals with *OPA1* variants.

To assess overall auditory decline, all available audiometric data points for probands were aggregated and plotted using better-ear PTA values (Figure 2). Linear regression confirmed age-dependent deterioration in hearing, yielding the equation HL (dB) = 0.706 × Age + 17.03 (R^2^ = 0.587). The corresponding coefficient of determination was R^2^ = 0.587, indicating that approximately 60% of the variance in hearing thresholds was explained by age. The fitted regression curve and its 90% confidence interval (shaded in red) illustrate the expected range of hearing trajectories while reflecting variability in progression patterns.

Among the six patients carrying missense variants, four demonstrated characteristics consistent with ANSD. JHLB-7354 showed severe impairment on ASSR testing, whereas ABR responses were absent in JHLB-2582, JHLB-11374, and JHLB-13305. All four patients exhibited preserved DPOAE responses. The variant identified in JHLB-13305 is a novel *OPA1* variant that is currently classified as a VUS. As additional symptoms, JHLB-11374 showed peripheral neuropathy, ataxia, and external ophthalmoplegia.

### 3.3. Intervention

Six of eight individuals had used HAs, and satisfaction data were available for five. Among these five cases, four were unable to continue using HAs due to discomfort during daily use and insufficient improvement in hearing and communication. One of these individuals, JHLB-1912, eventually underwent CI. This patient experienced rapid progression from moderate to profound HL in the left ear, followed by deterioration of the right ear to profound HL within three months. At the age of 72 years, the patient received left-sided CI. Speech perception testing using the Japanese monosyllable, word, and sentence perception tests (iCI-2004) was administered before and at six months after CI, demonstrating favorable outcomes, with over 90% correct in quiet conditions on the Japanese word perception test (Table 3). Given the favorable auditory performance and high satisfaction with the first implant, the patient underwent CI in the right ear nine months later. The second implant yielded a similarly favorable speech perception outcome (Table 3).

Better-ear PTA thresholds (defined as the lower of the right and left ear PTA values at 0.5, 1, 2, and 4 kHz) are plotted against age at testing. Gray crosses indicate individual audiometric evaluations. The red line represents the linear regression model (HL [dB HL] = 0.706 × Age [years] + 17.03), and the shaded area denotes the 90% confidence interval. The vertical axis is displayed in audiogram style, with better thresholds at the top (0 dB HL). This figure illustrates a general trend of progressive deterioration of hearing with age and marked inter-individual variability (R^2^ = 0.587).

Scores (% correct) on Japanese monosyllable, word, and sentence tests were obtained before and at six months after cochlear implantation. Scores before implantation were measured with hearing aids. The iCI2004, a Japanese cochlear implant speech test battery, was administered at a presentation level of 65 dB SPL. “Silent” indicates testing in quiet, and “SN+10” indicates testing at a signal-to-noise ratio of +10 dB.

## 4. Discussion

### 4.1. OPA1 Variant Spectrum in Hearing Loss

*OPA1* is known to be a highly mutationally diverse gene. According to ClinVar (https://www.ncbi.nlm.nih.gov/clinvar/ accessed 18 November 2025), 415 unique *OPA1* variants have been reported as likely pathogenic or pathogenic, including 116 frameshift variants, 67 missense variants, 72 nonsense variants, and 77 splice-site variants, among others [32]. As shown in Table 1, we identified a total of eight *OPA1* variants. Among the five missense variants, one was pathogenic, two were likely pathogenic, and two were VUS. The remaining three variants were splice-site variants, all classified as VUS.

In a previous report, the DOA-plus phenotype is predominantly associated with missense variants in the GTPase domain of *OPA1* [8]. These missense variants are thought to exert a dominant-negative effect due to an abnormal protein that interferes with normal mitochondrial function. Missense variants are also often associated with the ANSD phenotype, whereas truncating variants, including frameshift, nonsense and splice-site variant in the *OPA1* that lead to haploinsufficiency, have not been associated with ANSD [13].

### 4.2. Clinical Findings

In this study, OA was observed in four of six patients, and all patients with pathogenic or likely pathogenic missense variants exhibited OA. *OPA1*-related disease may also manifest with isolated or predominantly auditory phenotypes, as previously reported by Milone et al. [33]. Previous studies have reported the penetrance of OA to be approximately 80–90%, while HL has been observed in 10–20% of affected individuals. Therefore, incomplete penetrance may have contributed to the absence of clinical manifestations in some carriers [34,35].

In our cohort, OA manifested in early childhood, whereas the age at onset of HL ranged widely from 6 to 60 years. In patients with pathogenic or likely pathogenic variants, HL onset was observed at 9 to 16 years of age. Yu-Wai-Man et al. reported that, in DOA-plus syndrome, vision loss usually begins in the first decade of life, whereas HL tends to manifest from late childhood to early adulthood. Therefore, *OPA1*-related HL is generally considered to have post-lingual onset [9]. However, the age at onset showed considerable variability in our cases, especially in patients carrying VUS variants. We also observed marked heterogeneity in the progression of HL. Six of the eight individuals showed progressive hearing deterioration, and one progressed rapidly to profound levels within a year. The patients with pathogenic or likely pathogenic variants showed progressive HL. This variability may be influenced by somatic mitochondrial DNA instability, as well as by the hypothesis proposed by Chao de la Barca et al. [36]. To evaluate the progression of HL, linear regression of hearing level in the better-ear against age was conducted (Figure 2). This analysis suggested a trend toward age-related hearing deterioration; however, given the small number of patients and heterogeneous longitudinal data, further investigation will be needed. This trend was also observed after excluding the audiometric data for one patient with rapid hearing deterioration.

Growing evidence indicates that some missense variants of *OPA1* lead to ANSD rather than HL of cochlear origin [10,13]. In this study, four of six patients with missense variants showed preserved outer hair cell function in which DPOAE responses were preserved and ABRs absent, or severe HL in ASSR, which is a hallmark of ANSD [11,12].

### 4.3. Intervention

It has been reported that many individuals with ANSD show limited functional hearing and poor speech understanding despite the use of HAs [37]. Previous reports indicate that missense variants in *OPA1* could present with an ANSD phenotype, whereas splice-site variants generally do not [13]. In our study, two of the three individuals with splice-site variants were unable to achieve adequate benefit from HAs. These findings suggest the potential for certain splice-site variants to be associated with cochlear–neural dysfunction resembling ANSD. In particular, individual JHLB-1912, who harbored a splice-site variant, showed poor word recognition using HAs, despite her pure-tone thresholds still being in the moderate range at that time. Although *OPA1*-related HL is often considered a form of ANSD, several reports have suggested that CI may be associated with favorable speech outcomes despite the neural origin of the deficit. In this study, the HL of individual JHLB-1912 progressed from moderate to profound. After receiving a CI, she demonstrated high word and sentence recognition scores (>90%) in quiet. Similar observations have been described by Santarelli et al. and Huang et al. In both reports, electrocochleography suggested dysfunction of distal auditory nerve dendrites, whereas electrical stimulation recruited preserved proximal axons and spiral ganglion cells, thereby re-establishing reliable neural transmission to the brain [7,13].

While HL has been reported in approximately 10–20% of individuals with *OPA1*-related OA in vision-centered cohorts, our results demonstrate that *OPA1* variants account for only a very small fraction (8 of 18,475; 0.043%) of cases in a large HL cohort. This asymmetry underscores how disease prevalence estimates for *OPA1* vary substantially depending on the clinical ascertainment strategy. Nonetheless, small sample size of *OPA1*-related HL patients, limited segregation data, and the predominance of VUS without functional analysis remain important limitations of this study. Larger, multicenter studies incorporating functional assays and refined genotype–phenotype correlations will be essential for clarifying pathogenicity, improving prognostic accuracy, and providing personalized management for *OPA1*-related HL.

## 5. Conclusions

This study aimed to clarify the prevalence and clinical characteristics of *OPA1*-related HL in a large HL cohort, demonstrating that *OPA1*-related HL is rare from an audiological perspective, accounting for eight probands of 18,475 (0.043%) cases. HL was typically post-lingual in onset and progressive, with predominantly mild-to-moderate severity. Affected individuals with VUS exhibited phenotypic heterogeneity, which may reflect the uncertain pathogenicity of these variants, incomplete penetrance, and the influence of mitochondrial component genes. Missense variants were frequently associated with the clinical features of OA and ANSD. In our study, a favorable CI outcome was observed in a patient with *OPA1*-related HL, supporting the results of previous reports. Our findings highlight the importance of genetic diagnosis and comprehensive management in individuals with *OPA1* variants.

## Figures and Tables

**Table 1:** All possibly pathogenic *OPA1* variants identified in this study (NM_015560).

Base Change	AA Change	Pathogenicity	ACMG	ToMMo 60K JPN Database	gnomAD v4.1	SIFT	PP2 HVAR	MutTaster	CADD Phred	REVEL	SpliceAI Δ Score	dbscSNV (ADA)	Reference
c.449-3C>G	-	VUS	PM2/PP3	-	-	-	-	-	-	-	AL 0.84	1.0000	This study
c.892A>C	p.Ser298Arg	LP	PM2/PM5_Strong/PP3	-	-	D	D	D	27.0	0/965	-	-	Ideura et al., 2019 [28]
c.1330G>C	p.Glu444Gln	VUS	PM2/PM5/PP3	-	-	D	D	D	28.4	0.859	-	-	This study
c.1334G>A	p.Arg445His	P	PP1_Strong/PM2/PP3	-	-	D	D	A	34	0.934	-	-	Shimizu et al., 2003 [29]
c.1635C>G	p.Ser545Arg	LP	PS1/PM2/PP1_Moderate/PP3	-	-	D	D	D	29.1	0.843	-	-	Hudson et al., 2008 [30]
c.2496+3A>G	-	VUS	PM2/PP3	-	-	-	-	-	-	-	DL 0.6	0.9998	This study
c.2708-4A>G	-	VUS	PM2/PP3	-	7.72 × 10^−7^	-	-	-	-	-	AG 0.81	0.9991	This study
c.2794C>T	p.Arg932Cys	VUS	PM2/PP3	-	1.11 × 10^−5^	D	D	D	34	0.855	-	-	Nochez et al., 2009 [31]

All variants are indicated according to NM_015560. AA, amino acid; P, pathogenic; LP, likely pathogenic; VUS, variant of uncertain significance; PP2, PolyPhen-2; SpliceAI Δ score shows the predicted splice site effect and maximum Δ score. AL, acceptor loss; DL, donor loss; AG, acceptor gain. dbscSNV (ADA) indicates the adaptive boosting scores. D (in SIFT), deleterious; D (in PP2), probably damaging; D (in MutTaster), disease causing; A, disease-causing automatic.

**Table 2:** Clinical characteristics of individuals with *OPA1* variants identified in this study (NM_015560).

Family No.	ID	Sex	Base Change	AA Change	OA	Other Symptoms	Age	Onset of HL	Configuration	Severity of HL	Progression of HL	ABR	ASSR	DPOAEs	Intervention	Discontinued HA Use
1	JHLB-1912	F	c.449-3C>G	.	N/A	Nothing	65	45	High	Severe	Y	N/A	N/A	Absent	CI	Y
2	JHLB-2582	M	c.892A>C	p.Ser298Arg	Y	N/A	34	16	Low	Moderate	Y	NR	N/A	Present	N/A	N/A
3	JHLB-13305	F	c.1330G>C	p.Glu444Gln	N/A	Nothing	23	18	Low	Normal	Y	NR	Moderate	Present	Hearing aid	Y
4	JHLB-7354	M	c.1334G>A	p.Arg445His	Y	N/A	9	9	Flat	Normal	N/A	N/A	Severe	Present	Hearing aid	Y
	Mother	F	c.1334G>A	p.Arg445His	Y	N/A	42	N/A	N/A	N/A	N/A	N/A	N/A	N/A	N/A	N/A
5	JHLB-11374	M	c.1635C>G	p.Ser545Arg	Y	Peripheral neuropathy, Ataxia, External ophthalmoplegia	67	10	Mid	Moderate	Y	NR	N/A	Present	Hearing aid	N/A
6	JHLB-4064	F	c.2496+3A>G	.	N	Nothing	22	6	Flat	Mild	N	N/A	N/A	N/A	Hearing aid	Y
	Father	M	c.2496+3A>G	.	N/A	N/A	55	30	High	Moderate	Y	N/A	N/A	N/A	None	N/A
7	JHLB-16789	M	c.2708-4A>G	.	N	Nothing	72	60	High	Moderate	N	N/A	N/A	N/A	None	N/A
8	JHLB-16179	F	c.2794C>T	p.Arg932Cys	N/A	N/A	49	30	High	Mild	Y	N/A	N/A	Absent	Hearing aid	N

F, female; M, male; OA, optic atrophy; Age, age at the time of genetic testing; Y, yes; N, no; N/A, data not available; Flat, flat audiometric configuration; High, high-frequency hearing loss; Mid, mid-frequency hearing loss; Low, low-frequency hearing loss; ABR, auditory brainstem response; ASSR, auditory steady-state response; DPOAEs, distortion product otoacoustic emissions; NR, no response. “Progression of HL” indicates whether the patient was aware of progressive hearing deterioration. “Discontinued HA use” indicates that the patient discontinued hearing aid use because the benefit was insufficient.

**Table 3:** Results of speech perception testing before and after cochlear implantation for JHLB-1912.

Speech Perception Tests		Right HA	Left HA	Right CI	Left CI
Silent	Monosyllable	0	0	66	61
	Word	0	0	92	96
	Sentence	0	0	95	100
S/N+10dB	Word	NA	NA	72	68
	Sentence	NA	NA	83	76

## Data Availability

The datasets used during the current study are available from the corresponding author upon reasonable request.

## Abbreviations

The following abbreviations are used in this manuscript:

ABR	Auditory brain stem response
ANSD	Auditory neuropathy spectrum disorder
CI	Cochlear implant
HL	Hearing loss
OA	Optic atrophy
SNHL	Sensorineural hearing loss
